# Conceptualisations of positive mental health and wellbeing among children and adolescents in low‐ and middle‐income countries: A systematic review and narrative synthesis

**DOI:** 10.1111/hex.13407

**Published:** 2021-12-14

**Authors:** Laoise Renwick, Rebecca Pedley, Isobel Johnson, Vicky Bell, Karina Lovell, Penny Bee, Helen Brooks

**Affiliations:** ^1^ Division of Nursing, Midwifery and Social Work, Faculty of Medicine, Biology and Health, School of Health Sciences University of Manchester Manchester UK

**Keywords:** children and young people, low‐ and middle‐ income countries, mental health, perceptions, wellbeing

## Abstract

**Background:**

Mental illnesses are the leading causes of global disease burden. The impact is heightened in low‐ and middle‐income countries (LMICs) due to embryonic care systems and extant barriers to healthcare access. Understanding children and adolescents' conceptualisations of mental health wellbeing in these settings is important to optimize health prevention and promotion initiatives.

**Objective:**

To systematically review and synthesize children and adolescents' conceptualisations and views of mental health and wellbeing in LMICs.

**Design:**

Ten databases were systematically searched from inception to July 2020 and findings from included studies were synthesized.

**Results:**

Twenty papers met eligibility criteria comprising qualitative, quantitative and mixed methods studies. Children and adolescents identified aspects of mental health and wellbeing, including positive affect and outlook and having sufficient personal resources to face daily challenges. Identified factors recognized the importance of activating both kin and lay networks in supporting and maintaining wellbeing. Conceptualisations of mental health and wellbeing were varied and influenced by culture, developmental stage and gender.

**Discussion and Conclusions:**

Irrespective of environmental and sociocultural influences on concepts of wellbeing and mental health, children and adolescents in LMICs can conceptualise these constructs and identify how they pursue positive mental health and wellbeing important for developing age and culture‐appropriate community mental health strategies. Our review highlights the need to extend inquiry to wider developmental stages and both across and within specific populations in LMICs.

**Patient and Public Involvement:**

Initial results were presented at stakeholder workshops, which included children, adolescents, parents and health professionals held in Indonesia in January 2019 to allow the opportunity for feedback.

## 1 INTRODUCTION

Mental health problems constitute the leading cause of disability among children and adolescents globally, accounting for almost half the disease burden.^1^ In childhood, they are linked with a myriad of social problems, such as substance misuse, academic failure and school drop‐out giving rise to impaired physical and mental health later in life.^2^, ^3^ There is a growing imperative to prevent and protect children and adolescents from developing mental illnesses as well as to promote positive mental health and wellbeing,^4^ particularly in low‐ and middle‐income countries (LMICs) where intervention implementation is hampered by limited healthcare resources.^5^

Research in school‐based mental health has focussed largely on reducing stigma and improving negative attitudes to encourage appropriate help‐seeking for mental health problems. As such, mental health literacy approaches have traditionally adopted a deficit approach.^6^ Evidence from systematic reviews and meta‐analyses shows that multicomponent mental health promotion interventions are effective,^7^, ^8^, ^9^ particularly when adopting a positive mental health stance rather than focusing exclusively on illness prevention.^7^, ^10^ There is conflicting evidence, however, and a separate review suggests that these interventions are minimally efficacious and do not demonstrate sustained effects in school‐going cohorts.^11^

Operationalizing and standardizing specific target outcomes related to positive mental health and wellbeing poses a distinct challenge.^11^ A range of relevant outcomes reflect the broad nature of the field and some are flawed due to ceiling effects in community‐based populations.^11^ Selecting multiple target outcomes for reviews increases the likelihood of achieving positive effects^10^ and highlights the need for greater clarity in concepts and outcomes measured. Additionally, in varied geographical settings, these constructs are likely to be influenced by the sociocultural contexts in which they are perceived. Few studies attempt to measure the success of community‐level promotion programmes to improve positive mental health and well‐being from the perspective of children and adolescents.^11^

There is increasing awareness that strengthening knowledge about what constitutes good mental health and how to maintain it may have a positive effect on overall wellbeing.^12^ Arguably, the success of health‐promoting initiatives is contingent on the extent to which interventions take into account an individual's own understanding and beliefs.^13^ Globally, there have been historical difficulties engaging and providing relevant services for children and young people (CYP). Preliminary evidence indicates comparable mental health‐promoting initiatives show promising effects in LMICs; however, there is a paucity of research in specific age groups and these programmes are limited to a small number of settings.^14^

Understanding child and adolescents' perspectives on what constitutes mental health and well‐being, and what they consider important determinants of these constructs, is necessary to aid in generating relevant, valid measures of user‐informed outcomes and optimizing community and population‐level interventions to reduce the global burden of youth mental illness. The aim of this study was to conduct a systematic review of empirical research evaluating the understanding, views and perspectives of children and adolescents about what constitutes positive mental health and wellbeing^15^ in LMIC settings. Specifically, we sought to understand how children and adolescents construct concepts of mental health and well‐being, formulate what determines their own mental health and utilize self‐help strategies to manage their own emotional wellbeing. Multiple reviews have been conducted examining the impact of specific exposures or interventions on children and young person's wellbeing.^9^ Reviews examining the concepts of wellbeing and positive mental health from the perspective of young people are presently lacking.

## 2 METHODS

### 2.1 Design

The objectives were to search diverse academic literature to identify and synthesize empirical research conducted in LMIC populations that examines child and adolescents' perspectives of

1. positive mental health and wellbeing,
2. beliefs about factors that hinder and facilitate mental health and wellbeing and
3. self‐help strategies identified as helpful for improving individual mental health.

The methods and results are presented in line with Preferred Reporting Items for Systematic Reviews and Meta‐Analyses (PRISMA) guidelines.^16^ The review protocol was developed and revised by the authors. The protocol is registered in PROSPERO (CRD42019122057 available from https://www.crd.york.ac.uk/prospero/display_record.php?ID=CRD42019122057).

### 2.2 Search strategy and study selection

Ten databases (PsycInfo, EMBASE, Medline [OVID], Scopus, ASSIA [ProQuest], SSCI, SCI [Web of Science] CINAHL PLUS, Social Sciences full text [EBSCO]) were systematically searched from inception to July 2020. Initial searches were completed in January 2019 and again in July 2020. The search strategy was originally developed in two databases and customized to each of the other databases searched. Controlled vocabulary was included as medical subject heading and relevant articles were assessed for keywords to optimize the identification of articles of interest for the final searches. Four key components were used to structure the search strategy using the population, intervention, comparison, outcome (PICO) framework: (1) mental health and wellbeing, (2) LMICs, (3) perceptions and (4) child and adolescent populations. In each component, synonyms were combined using the Boolean operator ‘OR’ and across components using ‘AND’. Searches were adapted as necessary for individual databases. An example search strategy is available from the author on request. Forward citation tracking was undertaken for included studies up to April 2020.

### 2.3 Eligibility criteria

This review included original research that utilized primary data to examine the conceptualisations, views and perceptions of children and adolescents regarding positive mental health and wellbeing. Studies that had used qualitative, quantitative or mixed‐method designs were included. Also, studies that reported on samples of children and adolescents in LMICs with a mean age under 18 were included. LMIC countries were defined by the Organization for Economic Cooperation and Development (OECD) Development Assistance Committee 2018–2020. Peer‐reviewed journal articles and dissertations were included. Conference paper authors were contacted to include peer‐reviewed, full‐text articles of studies where available. Non‐English studies were included, and data were extracted by bilingual researchers affiliated with the study team. No date restrictions were used, and studies were not excluded based on the results of the quality assessment. Full inclusion/exclusion criteria can be found in Table 1.

**Table 1:** Inclusion and exclusion criteria

Category	Inclusion	Exclusion
Population of interest	Views, attitudes and perceptions of under 18‐year old's towards mental health, emotional well‐being and treatment‐seeking for mental health problems (where children and young people are employed the mean age of the sample will be less than 18 years old). Data collected within a low‐/middle‐income country (as defined by OECD's DAC list for 2018–2020).	Data obtained representing the views of CYP, parents, teachers or other professionals where individual CYP data cannot be extracted. Data collected in high‐income countries employing ethnic minorities originating from low‐/middle‐income countries (as defined by OECD's DAC list for 2018 to 2020). Studies where the primary research question is about developmental disorders.
Study types and designs	Primary data from observational studies, cross‐sectional data, surveys, other nonexperimental quantitative research, and qualitative and mixed methods studies were included.	Not primary data. Data from reviews.
Health outcomes and outputs of interest	Perceptions and views to include knowledge, attitudes, beliefs about wellbeing and mental health. ‐ Knowledge and beliefs about conceptualisations of wellbeing and mental health. ‐ Knowledge and beliefs about strategies to enhance wellbeing. ‐ Knowledge and beliefs about. ‐ Factors that enhance or threaten wellbeing and mental health.	Studies that do not measure the outcome of interest.
Publication dates	All publication dates	
Publication languages	All languages	

Abbreviations: DAC, Development Assistance Committee; OECD, Organization for Economic Cooperation and Development.

### 2.4 Screening

**Figure 1:**
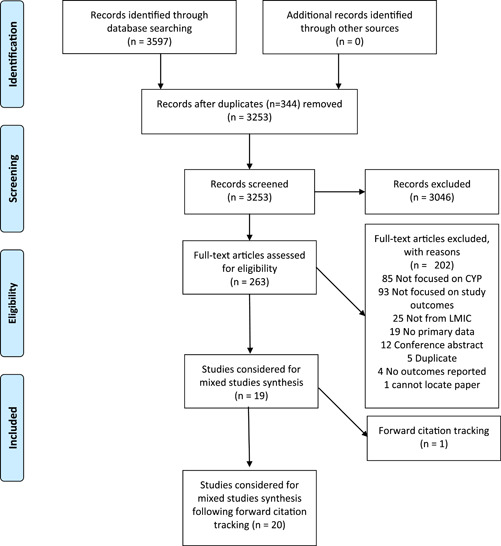
PRISMA flow diagram depicting flow of information screened and reviewed from: Moher D, Liberati A, Tetzlaff J, Altman DG, The PRISMA Group (2009). Preferred Reporting Items for Systematic Reviews and Meta‐Analyses: The PRISMA Statement. PLoS Med 6[7]: e1000097. doi:10.1371/journal.pmed1000097 for more information, visit www.prisma-statement.org. LMIC, low‐ and middle‐income countries; PRISMA, Preferred Reporting Items for Systematic Reviews and Meta‐Analyses

Returned records from database searches were combined, duplicates removed using Endnote software, and remaining references imported to the Covidence tool (https://www.covidence.org/). Two reviewers independently screened the title and abstracts of each study for relevance during the first stage of screening. Full texts of potentially relevant articles and those which did not contain sufficient information at the level of title and abstract were obtained and double‐screened by two reviewers. Disagreements regarding inclusion or exclusion that did not achieve consensus were resolved by a third reviewer not involved in the original decision. The authors met regularly throughout the review process to discuss the process of screening and resolve any difficulties or challenges in the process. Reasons for exclusion at the level of the full text are documented in the PRISMA diagram^16^ (please see Figure 1).

### 2.5 Data extraction

We extracted data using electronic forms created in Microsoft Excel expressly for the purpose of organizing data from selected studies. We piloted extraction using 10 studies across the review team to ensure field titles and descriptors were interpreted and extracted consistently allowing further iterations before extracting all data. Data extraction and quality assessments were conducted simultaneously by the study team (L. R., H. B. and R. P.).

Primary data were extracted from quantitative, qualitative and mixed methods studies relating to the review questions simultaneously. Data relating to study conduct were also extracted including publication, country, and setting (community, school‐based, clinical), study design, primary aim, metholodological approaches employed and other relevant contextual information.

### 2.6 Quality assessment

We anticipated a wide range of methodologies in this review signalling the need for a mixed methods appraisal approach and quality assessment of included studies was undertaken using the Mixed Methods Appraisal Tool (MMAT).^17^ We used the criteria corresponding to qualitative, quantitative descriptive and mixed methods designs based on the studies selected for review. Scores were expressed as a percentage of possible items divided by affirmative items. Each study was then classified as weak (≤50%), moderate–weak (51%–65%), moderate–strong (66%–79%) or strong (≥80%) based on a methodological scoring system.^18^ Quality was individually assessed by reviewers with 10% checked for accuracy. Any disagreements were resolved by discussion among reviewers. No records were excluded based on quality alone, but the quality assessment was used to inform the narrative synthesis of included studies.

### 2.7 Data analysis and synthesis

Due to the heterogeneity of included study designs and outcome measures, a narrative synthesis was used to synthesize data, which was guided by the Economic and Social Research Council (ESRC) guidance on the conduct of narrative synthesis.^19^ This was undertaken collaboratively between authors (L. R., H. B. and R. P.) and the resultant presentation of results was discussed among the wider study team. We used thematic analysis to map data to our three research questions deductively, arranging evidence with similar lines of evidence to ensure reliability. We then conducted analysis inductively to systematically generate theory about wellbeing and mental health in response to each question ensuring that each piece of information was relevant to the synthesis. Quantitative and qualitative data were synthesized simultaneously, and we used textual description, grouping and tabulation methods for preliminary synthesis and to explore patterns across studies. Included studies were tabulated in terms of study characteristics and extracted data. Initial inductive coding was undertaken at the point of extraction to characterize data in relation to descriptive categories. Differences in identified categories in relation to the country of origin, age and gender of included participants and other relevant contextual information were considered next. Finally, the draft synthesis was considered in light of the quality appraisal results (see below).

## 3 RESULTS

### 3.1 Study characteristics

A PRISMA study flow diagram in Figure 1 describes how studies were selected for inclusion in the review. A total of 20 papers describing 19 studies were included comprising 11 qualitative studies, 4 quantitative studies, primarily survey designs and 5 mixed methods studies. There were no experimental studies and one quasi‐experimental study that reported qualitative data relevant to our review question among the selected studies. A total of 22,257 children and adolescents were included across studies though most individuals (*n* = 17,854) came from one study that analysed the network structure of adolescent well‐being using a psychometrically tested tool in a representative sample across multiple sites across China.^20^ Quantitative evidence came from validation studies assessing the equivalence of a Swedish measure of mental health^21^ and novel psychological wellbeing scale development and validation.^22^ Sharma et al.^23^ used a rudimentary adaptation of the mental health literacy scale to evaluate help‐seeking preferences and priorities among Indian adolescents and Davids et al.^24^ used the health‐promoting lifestyle questionnaire to examine mental health behaviour and psychological wellbeing. There were no studies published in the least developed nations, 9 reported research conducted in upper middle‐income countries and 11 were conducted in lower middle‐income countries as per OECD classification. There were insufficient data to evaluate conceptualisations across WHO regions.

Participants in this review had a mean age of 16.6 (range: 6–26) and the majority were adolescent and young person populations. Four studies included children under the age of 10 alongside adolescents.^20^, ^25^, ^26^, ^27^ Apart from three studies,^28^, ^29^, ^30^ two of which reported on the same sample, all studies drew their sample from school‐going populations. Two studies were non‐English (Spanish and Portuguese) and data in these studies were extracted by affiliated researchers. Study characteristics are detailed in Table 2.

**Table 2:** Study characteristics and summarized findings

Reference, country and continent (author last name, year)	Study design (data collection methods)	Sample (*N*; *n*, % female)	Sample age (mean, [SD, age range])	Aim	Findings
Upper middle‐income countries					
Suttharangsee (1997) Thailand	Qualitative—ethnonursing	23; 13 (56%)	17 [N/R, N/R]^a^	To assess views about what constitutes mental health and beliefs about factors for achieving and maintaining positive mental wellbeing	Mental health good mood (smiling, being cheerful and polite and being worry‐free), positive thinking (good attitude towards one's self, focussing on the positive aspects of others and of situations) and good social relationships (being friends with others and ability to manage problems). Consistent with Thai cultural belief and Buddhism
Yu et al. (2019) China	Qualitative—multimethod comprising photovoice, community mapping and focus group discussions	90; 44 (48.8%)	17.4 [1.3; 15–19]	To understand the factors that facilitate and hinder disadvantaged adolescents from obtaining the health information and services they need to secure good health	Mental health perceived mainly in negative terms
					frustration, low self‐esteem, mood swings
Zeng et al. (2019) China	Survey design—longitudinal	17,854; 8306 (46.5%)	N/R [N/R; 6–18]	To analyse the network structure of adolescent well‐being and identify the central well‐being traits utilizing data from 11 samples of adolescents from primary and secondary schools in rural and urban areas of Southern, Northern and middle parts of China	Perceptions of wellbeing
					cheerfulness, engagement in current activity optimism for the future Engagement relates to the high value placed on achievement in Chinese culture
Davids et al. (2017) South Africa	Survey design—cross‐sectional	243; 131 (53.8%)	16.31 [0.4, N/R]	To examine the relationship between psychological wellbeing and mental health self‐help behaviour	Mental health behaviours for wellbeing (most to least used among children and adolescents)
					spiritual growth, interpersonal relations, stress management Having intrinsic and extrinsic goals were correlated with positive affect (PA) while mental health behaviour did not significantly predict PA in regression analysis
					No gender differences in goals, affect or mental health behaviours
Morais et al. (2012) Brazil In Portuguese	Survey design	1168; 619 (53%)	15.80 [1.68, 10–21]	To explore the concepts of mental health and wellbeing and understand self‐help strategies to improve wellbeing	Psychological wellbeing manifest in children and adolescents
					Positive attributes and behaviours (good relationships, happiness, positive thoughts, energy, equilibrium) Control (clear thoughts, life under control) Absence of illness (absence of maladaptive coping, no personal problems or illness, not seeing a therapist) Children and adolescents agreed on the importance of wellbeing and mind‐body connection
Nastasi and Borja (2015)^b^ Chapter 8—Perkins et al. Mexico	Mixed methods (some quantitative and some qualitative analysis—focus groups and ecomap activities)	68; 37 (54%) Focus group study 68; 52 (59%) Ecomap Study	N/R [N/R, 6–15] Focus group study 12 [N/R, 6–16] Ecomap Study	To explore stressors and sources of support for psychological wellbeing	Family most frequently occurring source of support, children and adolescents reported several sources of stress/negative influences on wellbeing:
					academics/school family, peers community Identified a range of positive self‐help strategies and females had a higher number of supportive relationships than males
Jenkins et al. (2019) Mexico In Spanish	Mixed‐methods comprising quantitative (sociodemographic questionnaire and standardized symptom scales) and qualitative (in‐depth ethnographic interviews, observation) components	35; 20 (57.1%)	15.9 [0.7, 15–17]	To generate an ethnographically informed understanding of contexts and processes that shape the emotional wellbeing and mental health of adolescents	Deficiencies in familial and close interpersonal relationships significant contributors to loneliness that lead to poor mental health
					A range of self‐help strategies identified, including music, exercise and sport, going online and spending time alone to process difficult emotions
Gonzalez‐Fuentez Palos (2016) Mexico In Spanish	Mixed methods comprising quantitative survey derived from qualitative analysis	1635; 856 (52.35)	N/R [N/R, 14–20]	To qualitatively evaluate the meaning of psychological wellbeing for adolescents and design and validate a scale to measure this construct	Factorial analysis of wellbeing components included seven factors:
					personal growth, positive relationships with others, purpose of life, self‐acceptance, plans for the future personal, rejection personal control
Nastasi and Borja (2015)^b^ Chapter 3—Lizardi and Carregari Brazil	Qualitative—multimethod comprising focus groups and ecomaps	55; 27 (49%)	N/R [N/R, 6–17]	To identify understandings of psychological wellbeing	Important sources of support identified included:
					parents, family relations, siblings, teachers Younger children were identified as having fewer self‐help strategies while older children were more likely to describe aggressive physical and verbal reactions as effective ways of coping with stress
Lower middle‐income countries					
Adelson et al. (2016)^c^ India	Qualitative—multimethod comprising focus groups, ecomap drawings and ecomap stories	37; 37 (100%)	N/R[12–20]	To explore perspectives of psychological wellbeing	Risk factors for poor psychological wellbeing occurred in the following domains:
					family/home system, friend system, intimate relationship system, school system, community system Self‐help strategies listed a number of activities and leveraging the support of others for advice and counsel
Nastasi and Borja (2015)^c^,^b^ Chapter 6—Adelson et al. India	Qualitative—multimethod comprising focus groups and ecomaps	37; 37 (100%)	N/R, [N/R, 12–20]	To explore stressors and protective factors for psychological wellbeing	Risk factors affecting wellbeing differed between females and males, the former reporting being less valued – maternal relationships were protective
Sharma et al. (2017) India	Survey design—cross‐sectional	354; 168 (47.5%)	N/R [N/R, 13–17]	To evaluate depression recognition, help‐seeking intentions and beliefs about interventions, causes, risk factors, outcomes and stigmatizing attitudes	Important sources of support identified included:
					exercise and sport, meditation
Parikh, Michelson et al. (2019) India	Qualitative—multimethod comprising stakeholder interviews and focus group discussions	191; 112 (58.7%)	N/R [N/R, 11–17]	To elicit the views of diverse stakeholders, including adolescents in two urban settings in India about their priorities and preferences for school‐based mental health services	Risk factors identified as targets for positive mental health strategies include:
					pressure to perform in exams, anxiety about securing a job after education, one‐sided romantic attractions, rejections and break‐ups in romantic relationships,
					gaining peer acceptance, bullying, peer pressure, family conflicts (often resulting from disagreements related to education and romantic relationships)
Shadowen et al. (2019) India	Mixed methods—quasi‐experimental design with qualitative inquiry	15; N/R (N/R)	N/R; [N/R, 12–14]	To measure the impact of an after‐school resilience‐building programme for a group of marginalized Indian school children in rural farming villages of Tamil Nadu, India	Risk factors identified females feeling less valued due to their gender status and children and adolescents repeatedly reported the value of meditation as a coping strategy
Nguyen et al. (2013) Vietnam	Qualitative—multimethod comprising stakeholder interviews, key informant interviews, focus groups	138; 83 (60%)	N/R [N/R, 15–18]	To explore perceptions of mental health and views about what are the risks for mental health problems alongside identifying stakeholder strategies to improve mental health	Risk factors for poor mental health arose under the following themes:
					academic pressures, problems related to pleasure‐seeking, problems with love and sex
Willenberg et al. (2020) Indonesia	Qualitative—focus group discussions	86; 41 (47.7%)	17 [N/R; 16–18]^d^	To understand conceptualisations and perceived determinants of mental health from the perspective of Indonesian adolescents	Positive mental health characteristics include:
					happiness, personal control (of problems, emotions, stress and personal limits), ability to socialize and interact with others, spirituality Signs of poor mental health include: inability to cope with contemporary pressures, poor social skills, fractured relationships, males associated poor mental health with interpersonal violence, females associated it with self‐harm
					A range of social, parental, peer, media and community factors were identified as risks for poor mental health
					Protective factors comprised positive emotions (optimism, hopefulness), positive relationships with family and friends, balanced lifestyle, civic participation, prayer and worship

Estrada, Nonaka et al. (2019) Phillipines	Mixed methods comprising quantitative (cross‐sectional survey) and qualitative (in‐depth interviews) components	183; 58 (33.9%) Study 1: 171, Study 2: 12	N/R [N/R, N/R]	To describe the prevalence of suicidal ideation and behaviours, attitudes towards suicide among adolescent learners in alternative education. Additionally, relationships between suicidal ideation, behaviours, participant characteristics, attitudes and alternative learning environment were evaluated	Children and adolescents identified leveraging support from church ministries and being involved in pastoral activities as a specific self‐help strategy to enhance mental health
Secor‐Turner et al. (2016) Kenya	Qualitative	64; 32 (50%)	16.2 [N/R, 12–26]	To evaluate perceived barriers and facilitators of health in a cultural context	Maintaining health is strongly linked with education from peers, socializing and having positive peers and supportive relationships with family, particularly parents.
					Protective factors included:
					self‐esteem, empowerment, spirituality Barriers to emotional health include feelings of: increased stress, hopelessness, isolation, rejection, anger, aggression Risks include
					teasing, not having access to supportive adults, school
Tamburrino et al. (2020) Kenya	Qualitative	7; N/R	N/R [N/R, 14–17]	To explore how youth stakeholders conceptualize mental illness, contributing factors and required supports for disadvantaged young people in Kenya	Positive mental health conceptualized as:
					balanced state of mind, positive state of mind/absence of stress, being free from obstacles that can affect a person physically, emotionally or cognitively (irrespective of having a mental illness), being able to make good judgements, able to manage problems, sense of wellbeing, adopt resilient and positive behaviours, Risk factors for negative wellbeing states
					distorted sense of self during the developmental stage of adolescence, general increase in negative emotions experienced by young people, lack of parental support
Glozah (2015) Ghana	Qualitative study using semi‐structured interview	11; 6 (54.5%)	16.86 [N/R, N/R]	To explore perspectives of interpersonal support for personal wellbeing	Wellbeing conceptualized as:
					ability to perform daily functions, mental strength, sense of vitality, ability to make critical decisions
					Risk factors for poor health and wellbeing:
					inadequate sleep, strictness, teasing, arguments and quarrelling with family, friends and teachers Protective factors
					advice and encouragement from others, maternal advice to deal with interpersonal conflict, paternal advice on health‐promoting behaviours, religiosity and spirituality

Abbreviation: NR, not reported.

^a^ Mode reported.

^b^ Book Chapter.

^c^ Using the same data.

^d^ Median and/or IQR reported.

^e^Proportions reported in age bands.

### 3.2 Quality appraisal

The quality of studies was varied and scores on the MMAT ranged from 0% to 100%. All but one of the qualitative studies achieved a score of 80%–100%, indicating these studies were of high quality. Fewer quantitative studies achieved high scores within their category of assessment with half showing a risk of measurement and response bias. The quality of mixed methods studies was also varied and few of the mixed methods designs addressed issues of divergence between the findings from quantitative and qualitative methodologies, nor did they adequately identify the explanatory or exploratory nature of the chosen design. We extracted qualitative data from mixed methods studies; with the exception of one study [22] none of the available studies provided quantitative data relating to the research questions. The few cross‐sectional studies that were included were examined for their contribution to the synthesis to determine the weight of evidence from these sources. Quality appraisals are detailed in Table 3.

**Table 3:** Quality appraisal

	Screen		Qualitative				Quantitative descriptive						Mixed Methods Score					(%)
	1	2	1.1	1.2	1.3	1.4	1.5	4.1	4.2	4.3	4.4	4.5	5.1	5.2	5.3	5.4	5.5	
Suttharangsee (1997)	**✓**	**✓**	**✓**	**✓**	**✓**	**✓**	**✓**											100
Lizardi et al. (2016)	**✓**	**✓**	**✓**	**✓**	**✓**	**✓**	**✓**											100
Adelson et al. (2016)	**✓**	**✓**	**✓**	**✓**	**✓**	**✓**	**✕**											80
Glozah (2015)	**✓**	**✓**	**✓**	**✓**	**✓**	**✓**	**✓**											100
Secor‐Turner et al. (2016)	**✓**	**✓**	**✓**	**✓**	**✓**	**✓**	**✓**											100
Nguyen et al. (2013)	**✓**	**✓**	**✓**	**✓**	**✓**	**✓**	**✓**											100
Adelson et al. (2016)	**✓**	**✕**	**✓**	**✕**	**✕**	**✕**	**✕**											20
Tamburrino et al. (2020)	**✓**	**✓**	**✓**	**✕**	**✓**	**✓**	**✓**											80
Parikh et al. (2019)	**✓**	**✓**	**✓**	**✓**	**✓**	**✓**	**✓**											100
Yu et al. (2019)	**✓**	**✓**	**✓**	**✓**	**✓**	**✓**	**✕**											80
Willenberg et al. (2019)	**✓**	**✓**	**✓**	**✓**	**✓**	**✓**	**✓**											100
Davids et al. (2017)	**✓**	**✓**						**✓**	**✓**	**✕**	**✕**	**✓**						60
Morais et al. (2012)	**✓**	**✓**						**✓**	**✓**	**✓**	**✓**	**✓**						100
Sharma et al. (2017)	**✓**	**✓**						**✓**	**✕**	**✕**	**✓**	**✕**						40
Zeng et al. (2019)	**✓**	**✓**						**✕**	**✓**	**✓**	**✕**	**✓**						60
Perkins et al. (2015)	**✓**	**✓**											**✓**	**✓**	**✓**	**✓**	**✓**	100
Jenkins et al. (2019)	**✓**	**✓**											**✕**	**✕**	**✕**	**✕**	**✕**	0
Estrada et al. (2019)	**✓**	**✓**											**✓**	**✓**	**✓**	**✕**	**✓**	80
Shadowen (2019)	**✓**	**✓**											**✕**	**✓**	**✕**	**✕**	**✕**	20
Gonzalez‐Fuentes et al. (2016)	**✓**	**✓**											**✓**	**✓**	**✓**	**✕**	**✓**	80

To evaluate the robustness of our synthesis, we examined the construction of meaning for concepts within the analysis contained in each of our research questions when (a) studies with the lowest quality score were removed and (b) studies with both moderate and low scores were removed. We then assessed the contribution of each individual piece of evidence to the consistency of descriptions within our synthesis to examine whether different pieces of information were compatible with the overall synthesis. We removed irregular pieces of information that were not congruous with our interpretations. Removing the results from low‐quality studies (*n* = 3) did not influence the synthesis and the removal of both weak and moderately weak quality studies (*n* = 6) demonstrated some bearing on the quantity of evidence that supported the synthesis. As such the synthesis findings were drawn mainly from interpreting and integrating the findings from well‐conducted and reported qualitative studies. These are presented in Table 4.

**Table 4:** Summary of thematic analysis and quality

Study, year, country	Country	Quality score^a^	Research design	Research question	Theme
Suttharangsee 1997 Morais, Amparo et al. 2012 Glozah 2015 Gonzalez‐Fuentes and Palos 2016 Tamburrino, Getanda et al. 2018 Zeng, Peng et al. 2019 Willenberg, Wulan et al. 2020	Thailand Brazil Ghana Mexico Kenya China Indonesia	Strong Strong Strong Strong Strong Moderate‐weak Strong	Qualitative Quantitative Qualitative Mixed Methods Qualitative Quantitative Qualitative	Mental Health conceptualisation	Optimism
Suttharangsee 1997 Morais, Amparo et al. 2012 Glozah 2015 Tamburrino, Getanda et al. 2018 Zeng, Peng et al. 2019 Willenberg, Wulan et al. 2020 Gonzalez‐Fuentes and Palos 2016 Davids, Roman et al. 2017	Thailand Brazil Ghana Kenya China Indonesia Mexico South Africa	Strong Strong Strong Strong Moderate‐weak Strong Strong Moderate‐weak	Qualitative Quantitative Qualitative Qualitative Quantitative Qualitative Mixed Methods Quantitative		Self‐agency
Suttharangsee 1997 Morais, Amparo et al. 2012 Glozah 2015 Tamburrino, Getanda et al. 2018 Willenberg, Wulan et al. 2020	Thailand Brazil Ghana Kenya Indonesia	Strong Strong Strong Strong Strong	Qualitative Quantitative Qualitative Qualitative Qualitative		Daily life functioning
Morais, Amparo et al. 2012 Tamburrino, Getanda et al. 2018	Brazil Kenya	Strong Strong	Quantitative Qualitative		Morality
Glozah 2015 Parikh, Michelson et al. 2019 Adelson, Nastasi et al. 2015 Adelson, Nastasi et al. 2016 Perkins, Wood et al. 2015 Secor‐Turner, Randall et al. 2016	Ghana India India India Mexico Kenya	Strong Strong Strong Weak Strong Strong	Qualitative Qualitative Qualitative Qualitative Mixed Methods Qualitative	Risk & protective factors	Interpersonal relationships‐mistreatment
Nguyen, Dedding et al. 2013 Parikh, Michelson et al. 2019 Shadowen 2018 Adelson, Nastasi et al. 2015 Adelson, Nastasi et al. 2016	Vietnam India India India India	Strong Strong Weak Strong Weak	Qualitative Qualitative Mixed Methods Qualitative Qualitative		Family conflict
Adelson, Nastasi et al. 2015 Perkins, Wood et al. 2015 Parikh, Michelson et al. 2019 Willenberg, Wulan et al. 2020	India Mexico India Indonesia	Strong Strong Strong Strong	Qualitative Mixed Methods Qualitative Qualitative		Societal stressors
Adelson, Nastasi et al. 2015 Adelson, Nastasi et al. 2016 Perkins, Wood et al. 2015 Tamburrino, Getanda et al. 2018 Parikh, Michelson et al. 2019 Willenberg, Wulan et al. 2020	India India Mexico Kenya India Indonesia	Strong Weak Strong Strong Strong Strong	Qualitative Qualitative Mixed Methods Qualitative Qualitative Qualitative		Academic pressures
Secor‐Turner, Randall et al. 2016 Tamburrino, Getanda et al. 2018 Parikh, Michelson et al. 2019 Suttharangsee 1997	Kenya Kenya India Thailand	Strong Strong Strong Strong	Qualitative Qualitative Qualitative Qualitative		Personal attributes
Suttharangsee 1997 Adelson, Nastasi et al. 2015 Willenberg, Wulan et al. 2020 Secor‐Turner, Randall et al. 2019 Glozah et al. 2015 Jenkins, Sanchez et al. 2019	Thailand India Indonesia Kenya Ghana Mexico	Strong Strong Strong Strong Strong Weak	Qualitative Qualitative Qualitative Qualitative Qualitative Mixed Methods		Family & peer support
Suttharangsee 1997 Adelson, Nastasi et al. 2015 Jenkins, Sanchez et al. 2019 Perkins, Wood et al. 2015 Sharma, Banerjee et al. 2017 Lizardi and Carregari, 2015 Davids, Roman et al. 2017 Estrada, Nonaka et al. 2017 Shadowen, 2018 Willenberg, Wulan et al. 2020	Thailand India Mexico Mexico India Brazil South Africa Philippines India Indonesia	Strong Strong Weak Strong Weak Strong Moderate‐weak Strong Weak Strong	Qualitative Qualitative Mixed Methods Mixed Methods Qualitative Qualitative Quantitative Mixed Methods Mixed Methods Qualitative	Self‐help strategies	Distraction techniques
Suttharangsee 1997 Adelson, Nastasi et al. 2015 Jenkins, Sanchez et al. 2019 Perkins, Wood et al. 2015 Sharma, Banerjee et al. 2017 Lizardi and Carregari, 2015 Davids, Roman et al. 2017 Estrada, Nonaka et al. 2017 Shadowen, 2018 Willenberg, Wulan et al. 2020 Glozah, 2015	Thailand India Mexico Mexico India Brazil South Africa Philippines India Indonesia Ghana	Strong Strong Weak Strong Weak Strong Moderate‐weak Strong Weak Strong Strong	Qualitative Qualitative Mixed Methods Mixed Methods Quantitative Qualitative Quantitative Mixed Methods Mixed Methods Qualitative Qualitative		Leveraging social support

^a^ Weak ≤ 50%, moderate–weak = 51%–65%, moderate–strong = 66%–79%, or strong ≥ 80%.

### 3.3 Conceptualisation of mental health and wellbeing

Ten studies evaluated adolescents' views about what constitutes positive mental health, psychological wellbeing or health, social and emotional wellbeing^20^, ^21^, ^22^, ^24^, ^25^, ^31^, ^32^, ^33^, ^34^, ^35^ and explored factors that influenced the relationships and construction of the meaning of wellbeing.^25^, ^31^

A range of factors was adduced by young people to explain what it means to have mental health and wellbeing. Optimism was described as a consistent marker of psychological wellbeing among qualitative and quantitative studies encompassing themes of positive thinking, cheerfulness, happiness and good mood. Evidence obtained from several transparent and systematic qualitative analyses showed that having a positive outlook, positive thoughts and emotions,^33^, ^34^, ^35^ cheerfulness and optimism^33^ were central to young people's notions of mental health and wellbeing. Quantitative evidence provided firmer evidence that having a positive outlook was prioritized over other features of mental health perceived by adolescents. Data from 17,854 randomly selected Chinese primary and secondary level students aged 6–18 provided longitudinal evidence that optimism is a central aspect of wellbeing for young people.^20^ Similarly, data from a sizeable Brazilian sample also ranked cheerfulness high on the importance of mental health constituents.^21^ Qualitative analyses from individual studies explicated the varying facets of personal situations through which demonstrating positivity pervaded life situations. Keeping the focus on the positive aspects of situations and of other people,^33^ positive interactions with others and having harmonious personal relationships are principal to concepts of well‐being.^21^, ^31^, ^33^ Optimism and positivity were also considered to mean how one relates to oneself, having a good attitude towards oneself^33^ and self‐acceptance^22^ were viewed as crucial for emotional wellbeing.

Older adolescents included in qualitative studies to assess views about what constitutes mental health in Indonesia, Kenya, Ghana and Thailand viewed daily functioning and performing expected roles inherent to definitions of mental health. Ably performing daily activities and tasks as a fundamental condition^31^ and coping with everyday challenges^31^, ^33^, ^34^, ^35^ were further emphasized as key indicators of being mentally healthy. Quantitative data supported this viewing functioning in a narrower sense, confined to academic performance. In this latter study, mental health concepts were obtained from 1168 Brazilian adolescents. These were sampled purposively from different socioeconomic sectors, using an adapted measure and ranking potential components of well‐being by importance.^21^

Self‐agency emerged as a pertinent constituent of mental health and wellbeing, comprising multiple facets, including personal attributes and competency for making decisions, having a central purpose to guide life decisions and being given the responsibility to make one's own decisions. Evidence drawn from both qualitative and quantitative studies illustrated that having the necessary personal attributes to make one's own choices leading to competent decision‐making and being granted the freedom to do so by others signified the key elements of autonomous behaviour. Quantitative survey data from 1635 Mexican adolescents sampled purposively showed both future plans and having a purpose in life were key factors characterizing psychological wellbeing.^22^ Quantitative data from South African students randomly sampled from rural areas showed that intrinsic life goals were significantly positively correlated with psychological wellbeing.^24^ Having the freedom and ability to make one's own choices about own actions and enterprise^22^, ^31^, ^35^ were highly valued among respondents in a large cohort of adolescents in Brazil. Survey data demonstrated that having personal control of life choices^21^ is an essential aspect of mental health explicated by in‐depth qualitative interviews extending control to having command of one's problems, emotions, stress and personal limits.^35^ Personal attributes for self‐agency included maintaining balance for good decision‐making linked with well‐being,^34^ being principled and showing moral awareness, behaving normally^21^ and being ‘sober‐minded’ in judgements.^34^

There were consistent findings that mental health was a significant concern and demonstrated clearly in qualitative analyses among samples of Indonesian, Vietnamese and Chinese adolescents^30^, ^32^, ^35^ and Indian adolescents advocating the need for school‐based mental health services.^36^ This was corroborated by quantitative data from Brazilian adolescents with the majority espousing the importance of mental health.^21^ Conversely, one study demonstrated adolescents in Kenya recognized that understanding of positive mental health was limited.^34^ Evidence from qualitative analyses consistently indicated that adolescent perspectives of mental health were often conflated with negative emotions and mental ill‐health and adolescents sometimes used others with mental illnesses as a frame of reference to describe their own views and perspectives of health states.^25^, ^30^, ^32^, ^35^

There was also some discrepancy about whether adolescents believed that to attain wellbeing, mental illnesses must be absent. For example, qualitative inquiry of Kenyan adolescents views of mental health and illness showed they believed one could possess attributes of good mental health while having a diagnosed mental illness.^34^ Quantitative data indicated the opposite and adolescents indicated that reliance on mental health professionals was contrary to attaining good mental health.^21^

### 3.4 Risk and protective factors for mental health and wellbeing

Further to conceptualisations of positive mental health, 12 studies investigated factors that lead to either positive or negative impacts on psychological, emotional wellbeing or mental health and protective factors for maintaining wellbeing.^25^, ^26^, ^27^, ^28^, ^29^, ^31^, ^33^, ^34^, ^35^, ^36^, ^37^, ^38^ Individual, school and community level factors were identified from the perspective of CYP in included studies.

The principal source of stress identified from qualitative analyses related mainly to interpersonal relations with close family and wider social networks. Strictness, teasing, arguments and quarrelling with family, friends and teachers were cited as a major source of stress and threat to wellbeing for adolescents.^31^, ^36^ Fighting with friends was a significant pressure identified in several reports in Ghana and India.^28^, ^29^, ^31^, ^36^ In terms of peer relations, a number of studies found that perceived mistreatment, including being teased, insulted, ignored, rejected or betrayed, negatively affected psychological wellbeing^25^, ^26^, ^31^ (Nastasi & Borja, 2015). More extreme concerns within wider peer networks were experiences of being isolated, excluded and bullied or even beaten and were perceived as negative impacts in both India and Kenya.^25^, ^28^

Family conflict and disagreements, particularly about education and romantic relationships were considerable wellbeing stressors.^32^, ^36^ Pleasure‐seeking was an identified risk factor among these children and adolescents, specifically becoming over‐involved in hedonistic pursuits could lead to delinquent behaviours like following media personalities, gaming, internet use and cigarette smoking. Young people also reported awareness of generally increasing negative emotions and distortions in their sense of self during adolescent years as a specific threat to their wellbeing.^34^

Academic pressure is viewed as a consistent and considerable source of stress among adolescents in qualitative analyses. With the exception of younger groups,^28^ students overwhelmingly reported academic overload, especially with homework, being unable to complete work and projects on time and exam pressure.^26^, ^29^, ^34^, ^35^, ^36^ Anxiety about securing a job once education was completed was particularly salient among older adolescents.^36^ Students also reported strictness, especially among teachers, reportedly fearing teachers' reactions to them, the effect on their confidence, ability to express themselves and ill‐treatment negatively affecting their reputation among peers.^31^, ^35^ Unfairness and cruelty were words associated with how teachers treated students.^35^

Gender differences were evident in qualitative data reporting specific factors affecting females wellbeing. There were relatively fewer data on males views and perspectives. One study constructively illuminated the complexities of specific socioeconomic environments but the analysis was limited to 37 females in one setting in India without comparison with males.^28^ A second qualitative analysis of 15 Indian adolescents corroborated that parents mistreatment of adolescent girls, in contrast to males, left them feeling less valued^37^ and they perceived a lack of fairness and equality that was significant for emotional wellbeing. Females, in Adelson et al.'s^28^, ^29^ study, faced higher expectations, having to assume caring responsibilities, such as looking after a sibling while parents were at work, in addition to schoolwork. Particularly after menarche, female freedom was restricted; being told how to behave by parents, siblings and especially older brothers^28^ and this formed a significant source of stress impacting wellbeing. Females feared being threatened, grabbed or even sexually assaulted on the streets.^29^ This impacted their family relationships and they experienced significant fear, anger and embarrassment as a result.^28^ Specifically, they would be blamed or held responsible by others, especially the men in their families, if they were victims of such an attack in public.

Fewer studies addressed the question about which factors protect children and adolescents mental health and wellbeing. While interpersonal difficulties comprised the principal threat to wellbeing among selected studies, the support received through these relationships was reported to safeguard against developing emotional problems in several qualitative studies.^29^, ^33^, ^35^ Relationships with peers and family, particularly parents, were viewed as important; children and adolescents perceived promoting and maintaining social wellbeing as strongly linked with education from peers, socializing and having positive peer and supportive relationships with family, particularly parents.^25^ Effective coping during stressful times is not only closely linked with receiving positive support from others but also the quality of relationships becomes important. Family relationships were considered especially supportive if close family relations were both understanding and open‐minded^35^ and displayed warmth.^33^ Children and adolescents identified encouragement, advice, religious and spiritual support administered by close family and peers as key to managing stress.^31^ One study described how deficiencies in familial and close interpersonal relationships lead to the loneliness that contributed to poor mental health^38^ irrespective of the number of contacts in one's network.

Adolescents believed internal factors could potentially buffer against the ill effects of negative factors affecting mental health, such as having a spiritual mindset, feeling of being empowered or having self‐esteem.^25^, ^36^ Personal attributes were also important for maintaining wellbeing, such as self‐acceptance,^22^ being comfortable with oneself^34^ and taking pride in oneself.^33^ Overall, protective factors were understudied in the studies selected for this review and views about the origin and nature of psychological attributes were unexplored. Few wider societal factors were considered by adolescents to either improve or diminish one's wellbeing. Qualitative analyses in Indonesia, India and Mexico and quantitative estimates of stressors among 68 adolescents in Mexico found negative wider societal impacts were prominent and adolescents reported violence and crime were aspects significantly impinging mental health.^35^ Antisocial behaviour is a specific concern threatening adolescents safety, including theft and gun crime.^26^, ^29^

### 3.5 Self‐help strategies

Reported self‐help interventions incorporated both individual strategies and leveraging support from lay networks. One study looked at the relationship between such strategies and mental health outcomes and found that mental health behaviour was not a significant predictor of positive or negative affect.^24^ The majority described self‐help strategies and nonprofessional support sources alongside beliefs about the effectiveness of these.

Several self‐help strategies were considered important to managing positive mental health and promoting wellbeing; the most identified strategy was using distraction from stressful situations by engaging in valued activities. Activities identified included music,^26^, ^29^, ^33^, ^38^ exercise and sports,^23^, ^33^ watching television or reading a book,^26^, ^27^, ^38^ interacting with pets,^26^ prayer, religious worship or spiritual practices,^24^, ^33^, ^35^, ^39^ housework/cooking,^29^ gardening,^33^ meditation,^23^, ^37^ writing^33^ and going online.^38^ Overall, evidence to support the effectiveness of any individual strategy or comparative evaluations of different strategies were lacking and the evidence to support passive activities and recreation to enhance wellbeing is weak.

Children and adolescents in qualitative analyses identified the following groups as important sources of social support: parents^27^, ^29^, ^31^, ^33^ mothers in particular,^29^, ^31^ family members,^26^, ^28^, ^29^ peers,^26^, ^27^, ^28^, ^29^, ^31^, ^33^ siblings^27^, ^33^ teachers,^26^, ^27^, ^33^ church leaders^39^ and neighbours/community members.^26^, ^29^ Friends were considered particularly useful through their role in the facilitation of distraction from stressful situations by encouraging the valued activity described above.^27^ However, all identified sources of support were also considered to be potential sources of stress (see Section 3.4) highlighting the complexity of relationships with lay support networks for this group.^26^

In reporting preferences for self‐help strategies, differences relating to gender and age were limited in included studies. No differences were found between genders in stress response and self‐help choices to promote wellbeing in a qualitative analysis.^27^ This was supported by findings in another study comparing mental health management behaviours between genders using quantitative data relating to 243 South African adolescents.^24^ Two qualitative analyses provide evidence that for females, greater importance is placed on maternal relationships and the support and advice received through these bonds as well as having close female confidantes among other kin relations.^29^, ^31^ A separate study comprising 68 Mexican students found females had a higher number of supportive relationships than males but not a significantly higher proportion, indicating the size of males' wider supportive networks to be smaller when compared with female counterparts.^26^ Younger children were identified as having fewer self‐help strategies and older children were more likely to describe aggressive physical and verbal reactions as effective ways of coping with stress^27^ but investigation of any age differences that may exist were limited to this one study.

## 4 DISCUSSION

This review analysed and synthesized empirical evidence regarding beliefs and perceptions of children and adolescents about mental health and psychological wellbeing and, to our knowledge, is the first to do so among LMIC populations. Consistent with dominant conceptualisations in high‐income settings, children and adolescents in LMIC perceive wellbeing as a confluence of factors relating to having good feelings and emotions, functioning well on a daily basis and having sufficient personal resources, such as resilience and self‐esteem, to meet daily challenges.^40^, ^41^ However, we also identified differences, indicating that children's and adolescents' definitions of positive and mental wellbeing are likely influenced by both culture and developmental phases. Contrasted with youth wellbeing concepts in predominantly high‐income settings, there were divergent perspectives about the importance of individual attributes, such as body image and high‐risk behaviours, which did not feature in our synthesis.^41^ Wider conceptualisations from individual studies in this review demonstrated an emphasis towards explaining wellbeing and mental health using cultural and religious influences. This is consistent with theories that differences in values, self‐concept and relational beliefs influence how concepts of wellbeing and positive mental health are constructed and prioritized.^42^

This was equally evident in descriptions of how children and adolescents conceptualize self‐help in settings where models of self are linked with social harmony and construed through the lens of interpersonal relations as opposed to European–American models that emphasize personal independence.^43^ Children and adolescents explanations of mental wellbeing and protective factors, in our synthesis, reflected cultural and religious orientations. Our review found that mobilizing kin and lay networks, including community and religious leaders, is an important self‐help strategy in low resource settings. This is crucial knowledge for supporting the identification of community platforms for intervention as evidence shows the importance of community mental health strategies, which may have even greater relevance in low resource settings^44^ where scaling up mental health services and interventions have yet to be prioritized.^45^ Similarly, maintaining a narrow focus on individualized interventions could be an important missed opportunity, as family relationships and the supportive roles they assume, are more pronounced in LMICs reflecting collectivist cultural norms emphasizing family involvement in shaping understanding of how to obtain and maintain good mental health.^46^

The Lancet Commission on child health and wellbeing identifies that mental health problems are becoming dominant among this age group and substantial investment in prevention approaches is required.^45^ In LMICs, a particular focus on mental health literacy is recommended while broadening mental health literacy concepts to include positive mental health, which enhances the salience and applicability of this concept to this population group.^6^ A coherent discourse emerged that children and adolescents were able to conceptualize mental health attributes that transcend deficit‐based mental health concepts, the key to developing asset‐driven mental health promotion programmes and optimizing population‐level prevention and promotion interventions. Nonetheless, there are significant gaps in knowledge and research arising from this review. Encouragingly, the empirical evidence we included is relatively recent and increasing methodological quality demonstrating mental health and wellbeing research is gaining momentum. Still, evidence is concentrated on lower grade evidentiary research and while this provides a helpful exploration of child and adolescent perspectives and views, this synthesis is best regarded as indicative rather than definitive.

Substantial further research is required to develop and optimize culture‐specific population‐level prevention and promotion interventions. This synthesis represents research from just a few LMICs and is not representative of all children and adolescents in those countries or settings within countries. There were no studies from the least developed nations highlighting a significant gap in understanding of wellbeing perspectives to inform public health initiatives. Self‐help strategies were also under‐researched and some concentrated on useful strategies for one specific problem and minimal evidence to support themes identified as a whole. Exploring the range of mental health behaviours that enhance mental health, their relationship to mental health constituents and wellbeing is needed. Including an exploration of individual beliefs about the effectiveness of these strategies and potential ways to develop or enhance these for improving wellbeing would also be beneficial. One study demonstrated that stress management and health‐supporting behaviours were linked with wellbeing^24^ but empirical evidence in this area to support public health interventions is notably absent. Importantly, we found preliminary evidence of age differences in health beliefs, understanding of risk and protective factors and self‐help strategies for mental health and wellbeing. Adolescent development takes place within the context of family, peer, school and community environments that are underpinned by broader cultural influences that shape their views and perspectives.^45^ Further research is needed to understand wellbeing and mental health perspectives throughout all phases of childhood and adolescence, including the various context settings for individual psychological growth and a need for research that informs universal, yet targeted developmentally appropriate interventions.

### 4.1 Strengths and limitations

Children's and adolescents' understanding of how to obtain and maintain good mental health is understudied in both low‐ and high‐income settings^47^, ^48^ and there are significant gaps in the literature to contrast in different contexts. We did not exclude any study based on the quality, which may affect our interpretations; however, we employed robust systematic review methods, ensuring the integrity of the synthesis. We did not use an exhaustive list of search terms related to the phenomenon of mental health and wellbeing though we recognize that terms are used interchangeably in some circumstances and lack clarity and shared understanding such that research in this area can be difficult to synthesize. Our broad approach taken in our search strategy mitigates this to some degree. Nonetheless, the synthesis is dependent on existing evidence and as stated, there are significant gaps in research in these areas across LMICs. A further gap exists across demographic groups within the country. All but two studies researched school‐going populations and while these are important sites for delivering effective health promotion interventions,^9^, ^10^, ^14^ sizeable and varying portions of children and adolescent populations in low resource settings are absent from school or attend sporadically, thus this marginalized group are underrepresented in empirical evidence.

## 5 CONCLUSIONS

Mental illnesses are becoming dominant health problems of children and adolescents globally and LMICs, in particular, require actions to reduce barriers to achieving good mental health, including promotion of mental health literacy and stigma reduction. However, children and adolescent perspectives of mental health and wellbeing are poorly researched and understood within these settings. Our synthesis provides an indicative exploration of attributes that children and adolescents believe signify mental health and factors that support and impinge upon attaining good mental health. Our synthesis illustrates the need to consider promotion strategies that are framed positively to equip children and adolescents with the necessary personal resources and skills that are appropriate across developmental phases. Further research is required to explore mental health conceptualisations across and within LMICs with a particular emphasis on understanding mental health behaviours that support wellbeing.

## CONFLICT OF INTERESTS

The authors report no conflict of interest.

## AUTHOR CONTRIBUTIONS

Laoise Renwick, Rebecca Pedley and Helen Brooks drafted the manuscript; Laoise Renwick was responsible for organisation and preparation of the manuscript; Laoise Renwick, Rebecca Pedley, Isobel Johnson, Helen Brooks, Vicky Bell and Karina Lovell were responsible for the concept of the review and Penny Bee and Helen Brooks conceived the overall study from which the review was conducted; Isobel Johnson with oversight from Helen Brooks, Penny Bee, Rebecca Pedley and Karina Lovell developed and managed the search strategy; Laoise Renwick, Helen Brooks, Rebecca Pedley and Vicky Bell were responsible for data extraction and analysis was conducted by Laoise Renwick, Helen Brooks and Rebecca Pedley with input from the wider review team for analysis planning, interpretation of data and overall manuscript preparation.

## Data Availability

The authors confirm that the data supporting the findings of this study are available within the article and its Supporting Information Materials.
